# The SwissLipids knowledgebase for lipid biology

**DOI:** 10.1093/bioinformatics/btv285

**Published:** 2015-05-05

**Authors:** Lucila Aimo, Robin Liechti, Nevila Hyka-Nouspikel, Anne Niknejad, Anne Gleizes, Lou Götz, Dmitry Kuznetsov, Fabrice P.A. David, F. Gisou van der Goot, Howard Riezman, Lydie Bougueleret, Ioannis Xenarios, Alan Bridge

**Affiliations:** ^1^Swiss-Prot Group, SIB Swiss Institute of Bioinformatics, CMU, 1 rue Michel-Servet, CH-1211 Geneva 4, Switzerland,; ^2^Vital-IT, SIB Swiss Institute of Bioinformatics, Quartier Sorge, Bâtiment Génopode, CH-1015 Lausanne, Switzerland,; ^3^Bioinformatics and Biostatistics Core Facility, École Polytechnique Fédérale de Lausanne, CH-1015 Lausanne, Switzerland,; ^4^SIB Swiss Institute of Bioinformatics, CH-1015 Lausanne, Switzerland,; ^5^Global Health Institute, École Polytechnique Fédérale de Lausanne, Station 19, CH-1015 Lausanne, Switzerland,; ^6^Department of Biochemistry, University of Geneva, CH-1211 Geneva, Switzerland,; ^7^Switzerland National Centre of Competence in Research “Chemical Biology”, University of Geneva, CH-1211 Geneva, Switzerland and; ^8^Centre for Integrative Genomics, University of Lausanne, CH-1015 Lausanne, Switzerland

## Abstract

**Motivation:** Lipids are a large and diverse group of biological molecules with roles in membrane formation, energy storage and signaling. Cellular lipidomes may contain tens of thousands of structures, a staggering degree of complexity whose significance is not yet fully understood. High-throughput mass spectrometry-based platforms provide a means to study this complexity, but the interpretation of lipidomic data and its integration with prior knowledge of lipid biology suffers from a lack of appropriate tools to manage the data and extract knowledge from it.

**Results:** To facilitate the description and exploration of lipidomic data and its integration with prior biological knowledge, we have developed a knowledge resource for lipids and their biology—SwissLipids. SwissLipids provides curated knowledge of lipid structures and metabolism which is used to generate an *in silico* library of feasible lipid structures. These are arranged in a hierarchical classification that links mass spectrometry analytical outputs to all possible lipid structures, metabolic reactions and enzymes. SwissLipids provides a reference namespace for lipidomic data publication, data exploration and hypothesis generation. The current version of SwissLipids includes over 244 000 known and theoretically possible lipid structures, over 800 proteins, and curated links to published knowledge from over 620 peer-reviewed publications. We are continually updating the SwissLipids hierarchy with new lipid categories and new expert curated knowledge.

**Availability:** SwissLipids is freely available at http://www.swisslipids.org/.

**Contact:** alan.bridge@isb-sib.ch

**Supplementary information:** Supplementary data are available at *Bioinformatics* online.

## 1 Introduction

Lipids are a large and diverse group of biological molecules that perform a wide variety of biological functions (Sud *et al.*, 2007). They comprise the bulk of membrane bilayers (Holthuis and Menon, 2014; van Meer *et al.*, 2008) and energy stores (Lass *et al.*, 2011), provide markers for the recognition and sorting of distinct organelles (Balla, 2013), and regulate processes such as cell proliferation and death (Morad and Cabot, 2013), immunity (Knowlden and Georas, 2014; Simmons and Ishii, 2014), angiogenesis, cardiogenesis and neurogenesis (Mendelson *et al.*, 2014) and inflammation (Ji *et al.*, 2011). The lipid complement or ‘lipidome’ of individual cells may include hundreds of thousands of lipid structures whose occurrence is tightly regulated in response to changes in the cellular environment and lipid availability (Gross and Han, 2011; Shevchenko and Simons, 2010). Dysregulation of the lipidome is a feature of many pathological conditions including cardiovascular disease (Sigruener *et al.*, 2014), hypertension (Graessler *et al.*, 2009), diabetes (Meikle *et al.*, 2014) and Alzheimer (Bennett *et al.*, 2014; Mapstone *et al.*, 2014), for which lipids may comprise an important source of biomarkers. Pathogens also manipulate the host lipidome in order to facilitate their own persistence and replication (Loizides-Mangold *et al.*, 2014; Lyn *et al.*, 2014).

Elucidating the roles of lipids in biological systems requires the integration of quantitative measurements of lipidome composition with knowledge of lipid metabolic pathways, enzymes and interacting proteins. Lipidome composition may be analyzed using mass spectrometry (MS) and tandem mass spectrometry (MS/MS)-based approaches which are able to quantify hundreds of lipid species over a broad dynamic range (da Silveira Dos Santos *et al.*, 2014). These approaches provide information on the atomic composition of intact lipids and their component parts (such as acyl chains) which can be mapped to prior knowledge of lipid structures using a standardized hierarchical notation (Liebisch *et al.*, 2013). One of the foremost sources of such knowledge is the LIPID MAPS Structure Database (LMSD), which provides a comprehensive classification of over 37 000 lipid structures grouped in 8 major lipid categories—fatty acyls, glycerolipids, glycerophospholipids, sphingolipids, sterol lipids, prenol lipids, polyketides and saccharolipids (Fahy *et al.*, 2007). Other resources and tools for the analysis of lipidomic data include LipidBLAST, which provides a library of 120 000 lipid structures (some derived from LIPID MAPS) and associated theoretical tandem mass spectra (Kind *et al.*, 2013), LipidHome, a database of over 36 million hypothetical glycerolipid and glycerophospholipid structures specifically designed for MS data integration (Foster *et al.*, 2013) and Visualization and phospholipid identification (VaLID) which provides a search engine for a library of hypothetical glycerophospholipid structures somewhat similar to those of LipidHome (Blanchard *et al.*, 2013). Specialized software for annotating MS data include LipidXplorer (Herzog *et al.*, 2013), mzMine (Pluskal *et al.*, 2010) and Lipid Data Analyzer (Hartler *et al.*, 2011). A common feature of all these resources and tools is that they focus mainly on the classification and identification of lipids. Knowledge of lipid biology, including links to metabolic pathways and enzymes, may be found in public knowledge resources such as BRENDA (Chang *et al.*, 2015), HMDB (Wishart *et al.*, 2013), KEGG (Kanehisa *et al.*, 2014), MetaCyc (Caspi *et al.*, 2014) and Reactome (Croft *et al.*, 2014). Although KEGG has been used as basis for computational approaches for matching hypothetical structures to pathways (Yetukuri *et al.*, 2007), these knowledge resources do not aim to provide comprehensive coverage of the hundreds of thousands of lipids that may exist in nature.

Here, we describe the development of a new knowledge resource that is designed to help connect lipidomics and lipid biology—SwissLipids. Our aim in creating SwissLipids was to provide a comprehensive reference database that can be used to describe mass spectrometry-based lipid identifications and link them to curated knowledge of lipid structures, metabolic reactions, enzymes and interacting proteins. SwissLipids was created through an iterative process in which prior knowledge of lipid structures and metabolism curated from published literature is used to generate an *in silico* library of all feasible structures for common lipid categories. These structures form the basis for a hierarchical lipid classification that is consistent with currently accepted lipidomic data notation and the classification of LIPID MAPS. The result is a reference namespace for lipidomic data publication, data exploration and hypothesis generation, allowing users to ask questions such as ‘where has this lipid species been seen before?’, ‘what structures might this lipid species have?’, ‘what are the relevant metabolic reactions?’ and ‘what proteins are known to interact with these putative structures?’

## 2 Methods

### 2.1 Curation of lipid structures and metabolism

We performed systematic curation of experimental knowledge of lipid structures using the chemical ontology ChEBI (Hastings *et al.*, 2013) and of lipid metabolism using the annotated reaction database Rhea (Morgat *et al.*, 2015) for several of the most common lipid categories including glycerophospholipids, glycerolipids, fatty acyls and sphingolipids. We focused mainly on lipids and enzymes of *Homo sapiens*, *Mus musculus*, *Caenorhabditis elegans* and *Saccharomyces cerevisiae*, but information from other species was also included when required to complete metabolic pathways (as in the desaturation of polyunsaturated fatty acids or PUFAs). Enzymatic reactions were curated in the SwissLipids database by linking UniProtKB (UniProt Consortium, 2014) protein accession numbers to Rhea reaction identifiers. All annotations in SwissLipids are associated with supporting evidence which includes a mandatory evidence type, represented by a code from the Evidence Codes Ontology (ECO) (Chibucos *et al.*, 2014) and supporting source text and literature citation where applicable (articles from the scientific literature are represented as PubMed records). All annotations were checked for accuracy and consistency by a second curator.

### 2.2 Generation and annotation of lipid structures

Curated knowledge of experimentally characterized lipid structures and metabolism was used to design and create a library of theoretically feasible lipid structures *in silico*. Lipid structures were generated using the Java application SMILIB v2.0 (Schüller *et al.*, 2007) which combines SMILES (Weininger, 1988) representations of chemical substructures. Each structure was annotated with a standard name and abbreviations (created using a custom Perl script that follows accepted naming conventions of LIPID MAPS), as well as lipid class and fatty-acyl/alkyl components (including mappings to ChEBI) and their positions on the glycerol backbone. Formula, InChI and InChI key (Heller *et al.*, 2013) and masses were calculated using the MolConvert and cxcalc tools (http://www.chemaxon.com), and exact *m/z* values for the following selected adducts were also calculated: [M.] + , [M + H] + , [M + K] + , [M + Na] + , [M + Li] + , [M + NH4] + , [M − H] − , [M + Cl] − , [M + OAc] − . Mappings to corresponding structures in ChEBI, HMDB and LMSD were established by matching InChI keys.

### 2.3 Generation of the lipid hierarchy

Glycerolipid and glycerophospholipid structures were used as the basis for the generation of a hierarchy of analytical outputs from mass spectrometry experiments (Liebisch *et al.*, 2013). The lipid hierarchy was generated using custom software that takes as input a set of structures and a template file that specifies the required annotations for each of the corresponding higher levels and the relations that link them (Table 1). These annotations include lipid nomenclature and human readable descriptions, ChEBI identifiers for lipid parent classes and parent components, SMILES representations, formula, mass and *m/z* values for adducts, and InChI and InChI keys where applicable.

**Table 1.** The hierarchical classification used in SwissLipids

Level	Example
*Category*	Glycerophospholipid
*Class*	Glycerophosphocholine
*Class*	Monoalkylmonoacylglycerophosphocholine
*Species*	PC(O-36:5)
*Molecular subspecies*	PC(O-16:1_20:4)
*Structural subspecies*	PC(P-16:0/20:4)
*Isomeric subspecies*	PC(P-16:0/20:4(5Z,8Z,11Z,14Z))

The hierarchy includes seven levels that are illustrated below with a single example. The hierarchy is compatible with that of LipidHome (Foster *et al.*, 2013) but uses only known components in the generation of the base *Isomeric subspecies*. The hierarchy is fully mapped to ChEBI at all levels. The prefix ‘O−’ indicates an alkyl bond, the prefix ‘P−’ a 1*Z*-alkenyl bond and the absence of a prefix an ester bond. PC, phosphatidylcholine.

### 2.4 Curation of the lipid hierarchy

The lipid hierarchy was curated using experimental literature on the occurrence of lipids in biological systems. Qualitative annotations take the form of a link between a lipid identifier (generally one of *Species*, *Molecular subspecies* or *Structural subspecies*), a term from the Gene Ontology (GO) (Blake *et al.*, 2013) or the cross-species tissue and cell ontology Uberon (Mungall *et al.*, 2012), and a taxonomic identifier (NCBI Resource Coordinators., 2014). As with other annotation types, all annotations of lipid occurrence in SwissLipids are associated with supporting evidence represented by a code from the Evidence Codes Ontology (ECO) and supporting source text and literature citation where applicable.

### 2.5 Databases

SwissLipids data is organized in a set of MySQL tables. Data from external reference resources such as ChEBI (Hastings *et al.*, 2013), Rhea (Morgat *et al.*, 2015), UniProtKB (UniProt Consortium, 2014), GO (Blake *et al.*, 2013), Cellosaurus and Uberon (Mungall *et al.*, 2012) is sourced from local copies synchronized on a weekly basis.

### 2.6 Website development

The SwissLipids website has been developed using the AngularJS (http://www.angularjs.org) framework and server side PHP scripts (http://www.php.net). SVG images of molecules are generated from their *mol* representations using MolConvert (http://www.chemaxon.com).

## 3 Results and discussion

### 3.1 SwissLipids content

SwissLipids includes over 2000 curated enzymatic reactions which directly link more than 800 proteins (UniProtKB) and 1500 reactions (Rhea) and supporting evidence. These annotations cover a variety of lipid categories including glycerophospholipids, glycerolipids, sphingolipids, sterols, fatty acids, fatty alcohols and wax esters. As the number of experimentally characterized lipid structures constitutes only a small fraction of the number of possible structures that may exist in nature (Gross and Han, 2011; Shevchenko and Simons, 2010), the curated knowledge available in SwissLipids was used to design and generate an *in silico* library of feasible lipid structures for selected common lipid categories. The current version of this library features 244 155 structures—hereafter referred to as *Isomeric subspecies*—from 38 classes of glycerophospholipid and 13 classes of glycerolipid (Supplementary Table S1) including 80 fatty acids and 18 fatty alcohols (Supplementary Table S2). Forthcoming versions of SwissLipids will generalize this approach to other lipid categories for which curated data is available, including more classes of glycerophospholipid as well as sphingolipids. Structures are arranged in a hierarchical classification consistent with the latest lipid notation (Liebisch *et al.*, 2013) that includes 91 124 *Structural subspecies* (which assume knowledge of lipid class and the composition and positions of individual fatty acyl groups), 45 571 *Molecular subspecies* (which assume knowledge of lipid class and the composition of individual fatty acyl groups) and 5508 *Species* (which assume knowledge of lipid class and the sum composition of all fatty acyl groups) (Table 1). The SwissLipids hierarchy includes curated lipid identifications from published literature for over 300 *Species, Molecular subspecies and Structural subspecies*.

The combinatorial generation of lipid structures described here and elsewhere (Blanchard *et al.*, 2013; Foster *et al.*, 2013) may produce lipid structures that do not exist in nature, and SwissLipids includes significantly more glycerophospholipid and glycerolipid structures than reference databases such as LMSD (which leverages the expert knowledge of the LIPID MAPS consortium). Although the existence of these extra structures is currently unproven, the use of known structural building blocks from the reference ontology ChEBI ensures that they are at least relevant to our current understanding of lipid structures and metabolism, and the procedure for brute force enumeration of structures is completely transparent. The SwissLipids library also provides a means to link structures from other databases such as HMDB and LMSD to published lipid identifications and to metabolic reactions (described using Rhea) and enzymes (UniProtKB), and is cross-referenced by the experimental lipidomics platform of the LipidX consortium (www.lipidomes.org).

### 3.2 SwissLipids website and access

**Fig. 1.**
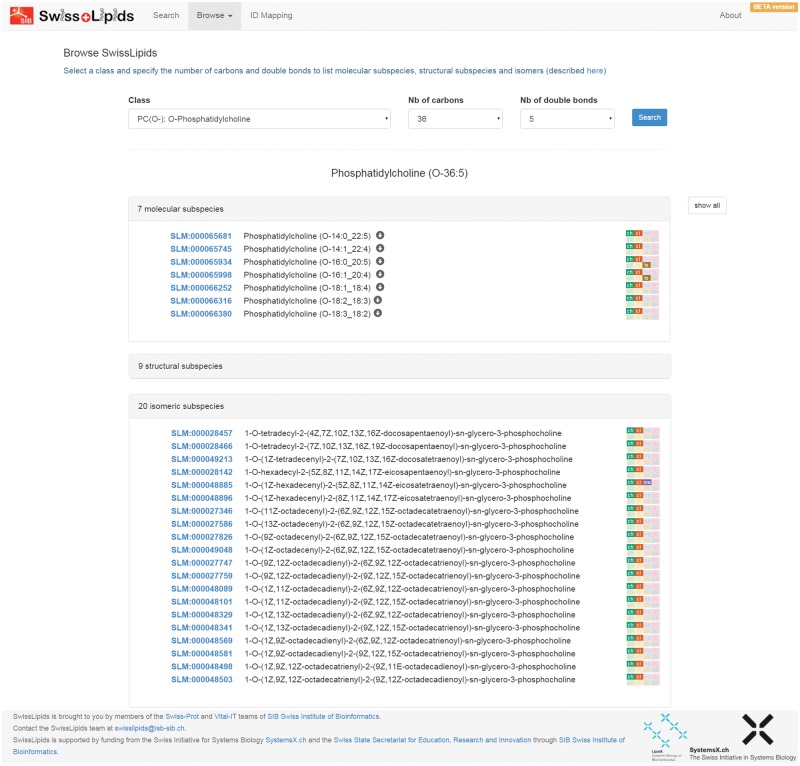
**Browsing SwissLipids**. Users can enter the lipid hierarchy at *Species* level by specifying the desired lipid class and number of carbon atoms and double bonds. The result lists consist of the corresponding *Molecular subspecies*, *Structural subspecies* and *Isomeric subspecies*. Color-coded icons with abbreviations provide an overview of the available information for lipids—their meaning can be revealed by moving the mouse over them. In this example the lipid Species Phosphatidylcholine (O-36:5) has 7 *Molecular subspecies*, 9 *Structural subspecies* (for which the list is not expanded) and 20 *Isomeric subspecies*. The *Molecular subspecies* Phosphatidylcholine (O-16:0_20:5) and Phosphatidylcholine (O-16:1_20:4) have experimental data on their location in specific tissues or taxa (indicated by the brown “lo” icon), while the *Isomeric subspecies* 1-*O*-(1*Z*-hexadecenyl)-2-(5*Z*,8*Z*,11*Z*,14*Z*-eicosatetraenoyl)-sn-glycero-3-phosphocholine has experimental data relating to metabolism (indicated by the purple ‘me’ icon). All lipids have cheminformatic descriptors (green ‘ch’ icon) and are classified (orange ‘cl’ icon) in the SwissLipids hierarchy

**Fig. 2.**
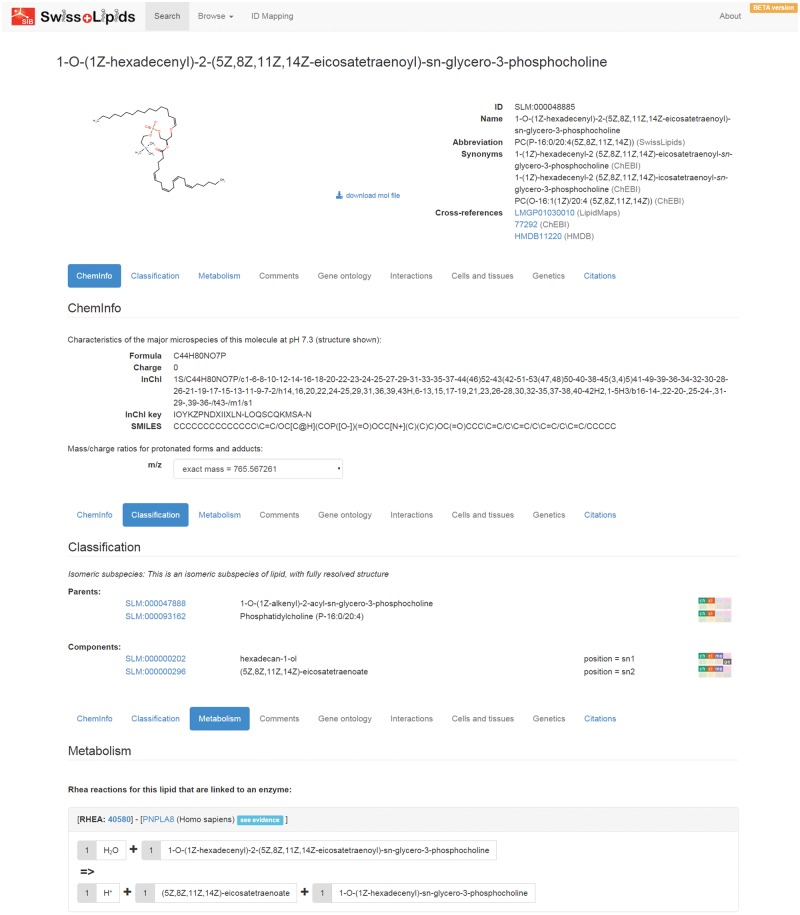
**A SwissLipids entry.** SwissLipids entry pages provide information on lipid structure and nomenclature (top panel), cheminformatics descriptors (second panel), lipid classification and components (third panel), reactions (Rhea) and enzymes (UniProtKB) (fourth panel), and subcellular (GO) and tissular (Uberon) location (not shown) which can be navigated through distinct tabs. Links to other databases such as ChEBI, HMDB and LIPID MAPS are also provided. The lipid classification indicates the structural class of lipid (here, 1-*O*-(1*Z*-alkenyl)-2-acyl-sn-glycero-3-phosphocholine) and its ‘analytical parent’ (the Structural subspecies Phosphatidylcholine (P-16:0/20:4)) as well as the individual components (hexadecanol-1-ol and 5*Z*,8*Z*,11*Z*,14*Z*-eicosatetraenoate) and the annotations available for each. The underlying evidence for curated assertions can be viewed by clicking on ‘see evidence’ (see fourth panel)

The SwissLipids website at www.swisslipids.org provides a simple interface to search for lipids (by identifiers, names, synonyms, abbreviations, formulae, *m/z* values for common adducts and SMILES, InChI and InChI key representations). Users may also browse the SwissLipids hierarchy at http://www.swisslipids.org/#/browse, beginning at *Species* level with the selection of the desired lipid class and the number of carbon atoms and double bonds in the acyl chains (Fig. 1). This returns a list of all possible *Molecular subspecies*, *Structural subspecies* and *Isomeric subspecies*. Exploration of the result lists is facilitated by a simple icon system that indicates the available information for each lipid entry (Fig. 1). Individual lipids as well as complete result lists may be selected for the identifier mapping service (http://www.swisslipids.org/#/mapper), which maps lipid identifiers from SwissLipids, ChEBI, HMDB and LMSD to each other as well as to the corresponding biochemical reactions from Rhea and curated enzymes from SwissLipids. Individual lipid entry pages provide key information including structure, cheminformatics descriptors, nomenclature, classification, reactions, enzymes, and subcellular and tissular location (Fig. 2). Enzyme names link to protein entry pages that list all curated information from SwissLipids, such as Rhea reactions and citations (not shown). SwissLipids may also be searched and accessed through the ExPASy bioinformatics resource portal (http://www.expasy.org) (Artimo *et al.*, 2012).

## 4 Conclusions

SwissLipids provides a core of curated information on lipid structures and metabolism which serves as the base for the generation of a library of all feasible lipid structures (according to our current knowledge of the relevant lipid structural classes) and their corresponding analytical outputs for MS and MS/MS. The curation of information using ChEBI, Rhea and UniProtKB provides a ready-made mapping of SwissLipids to these widely used resources and others that reference them such as the MetaboLights repository for metabolomic studies (Haug *et al.*, 2013), the IntEnz resource for enzyme nomenclature and classification (Fleischmann *et al.*, 2004), the MetaNetX.org platform for the analysis of metabolic models (Bernard *et al.*, 2014; Ganter *et al.*, 2013) and the genome annotation platform MicroScope (Vallenet *et al.*, 2013). As well as enriching SwissLipids with new lipid categories, structures and curated information, we plan to provide enhanced search options that include common isotopes and fragmentation patterns, and to improve interoperability by providing annotations and structures using the W3C standard RDF, a mainstay of large bioinformatics resources like UniProtKB.

## Supplementary Material

Supplementary Data
