# Supplementary material for: The SwissLipids knowledgebase for lipid biology
Source: Bioinformatics. 2015 May 5;31(17):2860–6. doi: 10.1093/bioinformatics/btv285 (PMC4547616; doi:10.1093/bioinformatics/btv285)
Supplement: Supplementary Data [file supp_31_17_2860__index.html]

The SwissLipids knowledgebase for lipid biology — The SwissLipids knowledgebase for lipid biology — Supplementary Data 

# The SwissLipids knowledgebase for lipid biology

## Supplementary Data

files

**Files in this Data Supplement:**

- Supplementary Data - docx file
