# Supplementary material for: The SwissLipids knowledgebase for lipid biology
Source: Bioinformatics. 2015 May 5;31(17):2860–6. doi: 10.1093/bioinformatics/btv285 (PMC4547616; doi:10.1093/bioinformatics/btv285)
Supplement: Supplementary Data [file supp_btv285_SwissLipids_bioinformatics_Table_S1_S2.docx]

**Table S1. Common glycerophospholipid and glycerolipid classes used for *in silico* structure generation.** Lipid class name, ChEBI identifiers and the number of resulting structures (Isomers) are indicated. Lipid classes marked “*” were curated into ChEBI during the course of this work.

| No. | Lipid class | ChEBI | Isomers |
| --- | --- | --- | --- |
| 1 | 1,2-diacyl-*sn*-glycero-3-phosphate | 58608 | 6,400 |
| 2 | 1-acyl-*sn*-glycero-3-phosphate | 57970 | 80 |
| 3 | 2-acyl-*sn*-glycero-3-phosphate | 64982 | 80 |
| 4 | 1-O-alkyl-2-acyl-*sn*-glycero-3-phosphate* | 73332 | 1,440 |
| 5 | 1-O-alkyl-sn-glycero-3-phosphate | 58014 | 18 |
| 6 | 1-O-(1Z-alkenyl)-2-acyl-*sn*-glycero-3-phosphate* | 77284 | 1,440 |
| 7 | 1-O-(1Z-alkenyl)-*sn*-glycero-3-phosphate* | 77283 | 18 |
| 8 | 1,2-diacyl-*sn*-glycero-3-phosphocholine | 57643 | 6,400 |
| 9 | 1-acyl-*sn*-glycero-3-phosphocholine | 58168 | 80 |
| 10 | 2-acyl-*sn*-glycero-3-phosphocholine | 57875 | 80 |
| 11 | 1-O-alkyl-2-acyl-*sn*-glycero-3-phosphocholine | 58666 | 1,440 |
| 12 | 1-O-alkyl-*sn*-glycero-3-phosphocholine* | 30909 | 18 |
| 13 | 1-O-(1Z-alkenyl)-2-acyl-*sn*-glycero-3-phosphocholine* | 77286 | 1,440 |
| 14 | 1-O-(1Z-alkenyl)-*sn*-glycero-3-phosphocholine* | 77287 | 18 |
| 15 | 1,2-diacyl-*sn*-glycero-3-phosphoethanolamine | 64612 | 6,400 |
| 16 | 1-acyl-*sn*-glycero-3-phosphoethanolamine | 64381 | 80 |
| 17 | 2-acyl-*sn*-glycero-3-phosphoethanolamine | 65213 | 80 |
| 18 | 1-O-alkyl-2-acyl-sn-glycero-3-phosphoethanolamine | 60520 | 1,440 |
| 19 | 1-O-alkyl-*sn*-glycero-3-phosphoethanolamine | 76168 | 18 |
| 20 | 1-O-(1Z-alkenyl)-2-acyl-*sn*-glycero-3-phosphoethanolamine* | 77290 | 1,440 |
| 21 | 1-O-(1Z-alkenyl)-*sn*-glycero-3-phosphoethanolamine* | 77288 | 18 |
| 22 | 1,2-diacyl-*sn*-glycero-3-phospho-(1’-sn-glycerol) | 64716 | 6,400 |
| 23 | 1-acyl-*sn*-glycero-3-phospho-(1’-sn-glycerol) | 64840 | 80 |
| 24 | 2-acyl-*sn*-glycero-3-phospho-(1’-sn-glycerol)* | 76528 | 80 |
| 25 | 1,2-diacyl-*sn*-glycero-3-phospho-1D-myo-inositol | 57880 | 6,400 |
| 26 | 1-acyl-*sn*-glycero-3-phospho-1D-myo-inositol | 64771 | 80 |
| 27 | 2-acyl-*sn*-glycero-3-phospho-1D-myo-inositol | 64872 | 80 |
| 28 | 1-O-alkyl-2-acyl-*sn*-glycero-3-phospho-1D-myo-inositol* | 78791 | 1,440 |
| 29 | 1-O-alkyl-*sn*-glycero-3-phospho-1D-myo-inositol* | 78793 | 18 |
| 30 | 1-O-(1Z-alkenyl)-2-acyl-*sn*-glycero-3-phospho-1D-myo-inositol* | 78792 | 1,440 |
| 31 | 1-O-(1Z-alkenyl)-*sn*-glycero-3-phospho-1D-myo-inositol* | 78794 | 18 |
| 32 | 1,2-diacyl-*sn*-glycero-3-phospho-L-serine | 57262 | 6,400 |
| 33 | 1-acyl-*sn*-glycero-3-phospho-L-serine | 64379 | 80 |
| 34 | 2-acyl-*sn*-glycero-3-phospho-L-serine | 65214 | 80 |
| 35 | 1-O-alkyl-2-acyl-*sn*-glycero-3-phospho-L-serine | 65220 | 1,440 |
| 36 | 1-O-alkyl-*sn*-glycero-3-phospho-L-serine* | 78790 | 18 |
| 37 | 1-O-(1Z-alkenyl)-2-acyl-*sn*-glycero-3-phospho-L-serine* | 78788 | 1,440 |
| 38 | 1-O-(1Z-alkenyl)-*sn*-glycero-3-phospho-L-serine* | 78789 | 18 |
| 39 | 1-acyl-*sn*-glycerol | 64683 | 51 |
| 40 | 2-acyl-*sn*-glycerol | 17389 | 51 |
| 41 | 3-acyl-*sn*-glycerol | 64760 | 51 |
| 42 | 1-O-alkyl-*sn*-glycerol | 15850 | 18 |
| 43 | 1-O-(1Z-alkenyl)-*sn*-glycerol* | 77297 | 18 |
| 44 | 1,2-diacyl-*sn*-glycerol | 17815 | 2,601 |
| 45 | 1,3-diacyl-*sn*-glycerol* | 77272 | 2,601 |
| 46 | 2,3-diacyl-*sn*-glycerol* | 75524 | 2,601 |
| 47 | 1-O-alkyl-2-acyl-*sn*-glycerol | 52595 | 918 |
| 48 | 1-O-(1Z-alkenyl)-2-acyl-*sn*-glycerol* | 77296 | 918 |
| 49 | 1-O-alkyl-3-acyl-*sn*-glycerol* | 77274 | 918 |
| 50 | triacyl-*sn*-glycerol | 64615 | 132,651 |
| 51 | 1-O-alkyl-2,3-diacyl-*sn*-glycerol* | 76585 | 46,818 |

**Table S2. Fatty acids and alcohols used for *in silico* structure generation.** Fatty acid/ alcohol name and ChEBI ID are indicated. Fatty acids and alcohols marked “*” were curated into ChEBI during the course of this work. Glycerophospholipid structures were created using all 80 fatty acids and alcohols. Glycerolipids were created using a subset of these (indicated by an entry the column “GL”) which excludes very long chain polyunsaturated fatty acids (VLC-PUFAs) over 24 carbons in length which have a restricted tissue distribution.

| No. | Fatty acid/alcohol | ChEBI | GL |
| --- | --- | --- | --- |
| 1 | acetate | 30089 |  |
| 2 | propionate | 17272 |  |
| 3 | butyrate | 17968 |  |
| 4 | hexanoate | 17120 |  |
| 5 | octanoate | 25646 |  |
| 6 | decanoate | 27689 |  |
| 7 | dodecanoate | 18262 | + |
| 8 | tridecanoate | 125832 | + |
| 9 | 11-methyldodecanoate* | 77197 | + |
| 10 | tetradecanoate | 30807 | + |
| 11 | (9Z)-tetradecenoate | 32370 | + |
| 12 | 13-methyltetradecanoate^*^ | 70826 | + |
| 13 | pentadecanoate* | 78795 | + |
| 14 | hexadecanoate | 7896 | + |
| 15 | (6Z)-hexadecenoate* | 76197 | + |
| 16 | (9Z)-hexadecenoate | 32372 | + |
| 17 | (9Z,12Z)-hexadecadienoate* | 77219 | + |
| 18 | 15-methylhexadecanoate* | 70838 | + |
| 19 | heptadecanoate | 32366 | + |
| 20 | octadecanoate | 25629 | + |
| 21 | (6Z)-octadecenoate | 32375 | + |
| 22 | (9Z)-octadecenoate | 30823 | + |
| 23 | (11Z)-octadecenoate | 30827 | + |
| 24 | (11E)-octadecenoate | 30828 | + |
| 25 | (9Z,12Z)-octadecadienoate | 30245 | + |
| 26 | (9Z,11E)-octadecadienoate | 17539 | + |
| 27 | (9Z,12Z,15Z)-octadecatrienoate | 32387 | + |
| 28 | (6Z,9Z,12Z)-octadecatrienoate | 32391 | + |
| 29 | (6Z,9Z,12Z,15Z)-octadecatetraenoate* | 77222 | + |
| 30 | nonadecanoate* | 78796 | + |
| 31 | eicosanoate | 32360 | + |
| 32 | (11Z)-eicosenoate | 32426 | + |
| 33 | (11Z,14Z)-eicosadienoate* | 77220 | + |
| 34 | (11Z,14Z,17Z)-eicosatrienoate* | 77223 | + |
| 35 | (8Z,11Z,14Z)-eicosatrienoate* | 71589 | + |
| 36 | (8Z,11Z,14Z,17Z)-eicosatetraenoate* | 71563 | + |
| 37 | (5Z,8Z,11Z,14Z)-eicosatetraenoate | 32395 | + |
| 38 | (5Z,8Z,11Z,14Z,17Z)-eicosapentaenoate* | 58562 | + |
| 39 | heneicosanoate* | 78797 | + |
| 40 | docosanoate | 23858 | + |
| 41 | (13Z)-docosenoate | 32393 | + |
| 42 | (13Z,16Z)-docosadienoate* | 77806 | + |
| 43 | (10Z,13Z,16Z)-docosatrienoate* | 82903 | + |
| 44 | (7Z,10Z,13Z,16Z)-docosatetraenoate* | 77225 | + |
| 45 | (7Z,10Z,13Z,16Z,19Z)-docosapentaenoate* | 77224 | + |
| 46 | (4Z,7Z,10Z,13Z,16Z)-docosapentaenoate* | 77226 | + |
| 47 | (4Z,7Z,10Z,13Z,16Z,19Z)-docosahexaenoate* | 77016 | + |
| 48 | tetracosanoate | 31014 | + |
| 49 | (15Z)-tetracosenoate | 32392 | + |
| 50 | (9Z,12Z,15Z,18Z)-tetracosatetraenoate* | 77227 | + |
| 51 | (9Z,12Z,15Z,18Z,21Z)-tetracosapentaenoate* | 77201 | + |
| 52 | (6Z,9Z,12Z,15Z,18Z)-tetracosapentaenoate* | 77228 | + |
| 53 | (6Z,9Z,12Z,15Z,18Z,21Z)-tetracosahexaenoate* | 77202 | + |
| 54 | hexacosanoate | 31013 | + |
| 55 | (17Z)-hexacosenoate | 77221 | + |
| 56 | (11Z,14Z,17Z,20Z)-hexacosatetraenoate* | 77229 |  |
| 57 | (11Z,14Z,17Z,20Z,23Z)-hexacosapentaenoate* | 77203 |  |
| 58 | (8Z,11Z,14Z,17Z,20Z)-hexacosapentaenoate* | 77230 |  |
| 59 | (8Z,11Z,14Z,17Z,20Z,23Z)-hexacosahexaenoate* | 77205 |  |
| 60 | octacosanoate | 31002 | + |
| 61 | (13Z,16Z,19Z,22Z)-octacosatetraenoate* | 77231 |  |
| 62 | (13Z,16Z,19Z,22Z,25Z)-octacosapentaenoate* | 77206 |  |
| 63 | (10Z,13Z,16Z,19Z,22Z)-octacosapentaenoate* | 77243 |  |
| 64 | (10Z,13Z,16Z,19Z,22Z,25Z)-octacosahexaenoate* | 77209 |  |
| 65 | triacontanoate | 31004 | + |
| 66 | (15Z,18Z,21Z,24Z)-triacontatetraenoate* | 77232 |  |
| 67 | (15Z,18Z,21Z,24Z,27Z)-triacontapentaenoate* | 77210 |  |
| 68 | (12Z,15Z,18Z,21Z,24Z)-triacontapentaenoate* | 77246 |  |
| 69 | (12Z,15Z,18Z,21Z,24Z,27Z)-triacontahexaenoate* | 77211 |  |
| 70 | (17Z,20Z,23Z,26Z)-dotriacontatetraenoate* | 77233 |  |
| 71 | (17Z,20Z,23Z,26Z,29Z)-dotriacontapentaenoate* | 77212 |  |
| 72 | (14Z,17Z,20Z,23Z,26Z,29Z)-dotriacontahexaenoate* | 77213 |  |
| 73 | (19Z,22Z,25Z,28Z)-tetratriacontatetraenoate* | 77234 |  |
| 74 | (19Z,22Z,25Z,28Z,31Z)-tetratriacontapentaenoate* | 77214 |  |
| 75 | (16Z,19Z,22Z,25Z,28Z,31Z)-tetratriacontahexaenoate* | 77215 |  |
| 76 | (21Z,24Z,27Z,30Z)-hexatriacontatetraenoate* | 77235 |  |
| 77 | (21Z,24Z,27Z,30Z,33Z)-hexatriacontapentaenoate* | 77216 |  |
| 78 | (18Z,21Z,24Z,27Z,30Z,33Z)-hexatriacontahexaenoate* | 77217 |  |
| 79 | (23Z,26Z,29Z,32Z)-octatriacontatetraenoate* | 77236 |  |
| 80 | (23Z,26Z,29Z,32Z,35Z)-octatriacontapentaenoate* | 77218 |  |
| 81 | 11-methyldodecan-1-ol* | 77438 | + |
| 82 | tetradecan-1-ol* | 77417 | + |
| 83 | 13-methyltetradecan-1-ol* | 77435 | + |
| 84 | hexadecan-1-ol | 16125 | + |
| 85 | 15-methylhexadecan-1-ol* | 77434 | + |
| 86 | octadecan-1-ol | 32154 | + |
| 87 | eicosan-1-ol* | 75627 | + |
| 88 | docosan-1-ol | 31000 | + |
| 89 | tetracosan-1-ol* | 77413 | + |
| 90 | hexacosan-1-ol | 28415 | + |
| 91 | octacosan-1-ol | 28243 | + |
| 92 | triacontan-1-ol | 28409 | + |
| 93 | (9Z)-octadecen-1-ol* | 73504 | + |
| 94 | (11Z)-octadecen-1-ol | 50405 | + |
| 95 | (13Z)-octadecen-1-ol* | 78805 | + |
| 96 | (11Z)-eicosen-1-ol* | 77412 | + |
| 97 | (13Z)-docosen-1-ol* | 77416 | + |
| 98 | (9Z,12Z)-octadecadien-1-ol* | 73534 | + |
|  |  |  |  |
